# Liver Graft MicroRNAs Expression in Different Etiology of Acute Jaundice after Living Donor Liver Transplantation

**DOI:** 10.3390/biology11081228

**Published:** 2022-08-17

**Authors:** Shu-Hsien Lin, Kun-Ta Wu, Chih-Chi Wang, Kuang-Tzu Huang, Kuang-Den Chen, Li-Wen Hsu, Hock-Liew Eng, King-Wah Chiu

**Affiliations:** 1 Division of Hepato-Gastroenterology, Department of Internal Medicine, Kaohsiung Chang Gung Memorial Hospital, Kaohsiung 83301, Taiwan; 2 Liver Transplantation Program, Kaohsiung Chang Gung Memorial Hospital, Kaohsiung 83301, Taiwan; 3 Division of General Surgery, Department of Surgery, E-Da Hospital, Kaohsiung 83301, Taiwan; 4 Division of General Surgery, Department of Surgery, Kaohsiung Chang Gung Memorial Hospital, Kaohsiung 83301, Taiwan; 5 College of Medicine, Chang Gung University, Taoyuan 33302, Taiwan; 6 Institute for Translational Research in Biomedicine, Kaohsiung Chang Gung Memorial Hospital, Kaohsiung 83301, Taiwan; 7 Department of Pathology, Kaohsiung Chang Gung Memorial Hospital, Kaohsiung 83301, Taiwan

**Keywords:** liver transplantation, microRNA, acute rejection, acute jaundice, liver biopsy, liver graft pathology

## Abstract

**Simple Summary:**

Acute jaundice, a critical problem after living donor liver transplantation, often required efforts to discriminate its etiologies. MicroRNAs are small non-coding RNAs, which express differently during disease development. To differentiate the etiology of acute jaundice after living donor liver transplantation, we examined hepatic microRNA expression patterns in several liver graft pathologies. Eighty liver transplant recipients undergoing post-transplant liver graft biopsy for the evaluation of acute jaundice were enrolled in this one-year prospective study. Using real-time quantitative reverse transcription-polymerase chain reaction profiling assay, we identified intra-graft microRNA (microRNA-122, microRNA-301, microRNA-133a, and microRNA-21) signatures in various allograft pathologies (37 recipients with acute cholangitis, 20 recipients with acute rejection, 12 recipients with recurrent hepatitis, 6 recipients with non-specific pathological change and 5 recipients with fatty change). In acute cholangitis, intra-graft microRNA-122, microRNA-301, and microRNA-21 significantly down-regulated; in acute rejection, intra-graft microRNA-122 significantly up-regulated and intra-graft microRNA-133a significantly down-regulated; in recurrent hepatitis, intra-graft microRNA-122, microRNA-301, and microRNA-21 remarkably up-regulated; and in fatty change, only intra-graft microRNA-133a up-regulated in significance. Our study suggests that specific intra-graft microRNA expression patterns as a checklist may be helpful for differential diagnosis of acute jaundice etiologies following living donor liver transplantation.

**Abstract:**

**Background:** Acute jaundice remains a critical problem following liver transplantation. MicroRNAs (miRNAs) are involved in regulating gene expression related to various disease phenotypes and statuses. **Aims:** To differentiate acute jaundice etiology after living donor liver transplantation (LDLT), we examined the hepatic miRNA expression patterns in several liver graft pathologies. **Methods:** Eighty liver transplant recipients undergoing post-LDLT graft biopsy for the evaluation of acute jaundice were enrolled in this 1-year prospective study. Using a real-time quantitative reverse transcription-polymerase chain reaction profiling assay, we identified hepatic miRNA (miRNA-122, miRNA-301, miRNA-133a, and miRNA-21) signatures in various allografts pathologies. **Results:** Pathologic findings of the 80 recipients were as follows: acute cholangitis (AC), 37 (46%); acute rejection (AR), 20 (25%); recurrent hepatitis (RH), 12 (15%); non-specific pathological change, 6 (8%); and fatty change (FC), 5 (6%). None of these identified hepatic miRNAs expression pattern was significantly correlated with serum parameters, including neutrophil-lymphocyte ratio. In AC, hepatic miRNA-122, miRNA-301, miRNA-133a, and miRNA-21 expression was significantly downregulated (*p* < 0.05). MicroRNA-122 expression was elevated in cases of AR and RH (*p* < 0.05); miRNA-301 and miRNA-21 expression was higher in RH than in AC (*p* < 0.05); and miRNA-133a expression was higher in FC than in AR (*p* < 0.05). **Conclusions:** Our study suggests that specific hepatic miRNA expression patterns as a checklist may be useful for differential diagnosis of acute jaundice following liver transplantation.

## 1. Introduction

In living donor liver transplantation (LDLT) settings, acute jaundice is a common and critical problem, resulting mainly from post-transplant acute rejection (AR) and recurrent hepatitis (RH), both of which are associated with an increased risk of graft failure or mortality [1]. Clinically, conventional serum liver biochemical tests, such as total bilirubin, alanine aminotransferase (ALT), aspartate transaminase (AST), alkaline phosphatase (ALK-P) and γ-glutamyl transferase (γ-GT), often lack sensitivity and specificity in current immune function monitoring [2]. Percutaneous liver graft biopsy is often warranted to establish a definite diagnosis of graft dysfunction [3]; however, serial biopsies are invasive and almost impractical in clinical practice [4]. Therefore, circulating miRNAs, either actively secreted from living cells or passively released by dying cells, have been implicated as promising biomarkers for detecting various liver pathologies and diagnosis post-transplant acute rejection [5,6].

MicroRNAs (miRNAs), approximately 22 nucleotides in length, are single-stranded noncoding RNA molecules that regulate the posttranscriptional expression of target genes by binding to complementary mRNAs at 3′-untranslated regions [7]. With the development of miRNA-profiling techniques, such as quantitative reverse transcription PCR (qRT-PCR), microarrays, and high-throughput next-generation RNA sequencing, researchers have extensively investigated the vital role of several identified or even previously unknown miRNAs as potential biomarkers for diagnosis, prognosis prediction, or evaluation of treatment response in various diseases, including cancer, cardiovascular disease, metabolic disease, and organ injury [8,9]. 

Changes in microRNA abundance and validated target genes during liver development, regeneration and disease were reported in the literature [10]; in fact, organ-specific microRNAs as potential biomarkers for monitoring immune rejection in organ transplantation would be a reflection of the complex and delicate interaction between the immunity and the liver allograft [11]. Recently, a pilot study conducted by Thangamani Muthukumar et al. demonstrated that circulating levels of miRNAs, quantified using customized RT-qPCR assays, may offer a rapid and noninvasive means of diagnosing AR in human liver allografts and for discriminating AR from intragraft inflammation or fibrosis due to recurrent HCV [12].

As reported in our previous study [13], the miR-122 expression level was significantly higher in native liver tissue than in plasma. In this current study, we identified miRNAs extracted from liver graft tissue, which may represent miRNA expression patterns more accurately than when plasma is used. MiRNA-122 is liver-specific [14], and hepatic miRNA-301 was reported be a biomarker of liver transplant rejection [15]. MiRNA-133a was uniquely expressed in cholangiocytes and the subepithelial compartment [16]. MiRNA-21 has also been associated with cholestasis, and its expression level increased during cholestatic injury [17,18].

Based on the aforementioned research, hepatocyte-specific and cholangiocyte-specific miRNAs might dysregulate in response to liver injury and cholestatic injury. In the setting of liver transplantation, differentiating the etiologies of acute jaundice via expression levels of the entire miRNome is challenging and difficult, and thus we would like to select and focus on the four specific miRNAs (miRNA-122, miRNA-301, miRNA-133a, and miRNA-21), which were reported to be in association with hepatocyte, cholangiocyte, and their dysfunction leading to post-LDLT acute jaundice.

We hypothesized that distinct liver graft miRNA expression patterns might serve as ancillary markers to discriminate the etiologies of post-LDLT acute jaundice. The aim of our study is to investigate the hepatic expression levels of miRNA-122, miRNA-301, miRNA-133a, and miRNA-21 in conjunction with liver graft pathology in patients with acute jaundice after LDLT.

## 2. Results

### 2.1. Patient Characteristics

The clinical characteristics of the 80 recipients are presented in Table 1. The mean age at transplantation was 52.56 (range: 20–66) years, and men constituted 72.5% of the population. Most cases of primary liver disease before LDLT were chronic hepatitis B (35%), chronic hepatitis C (27.5%), and alcoholic cirrhosis (17.5%). The mean serum total bilirubin level was 3.25 ± 6.81 mg/dL, and the mean neutrophil–lymphocyte ratio (NLR) was 3.39 ± 2.96.

### 2.2. Clinical Biochemistry Data and Liver Graft Pathologies 

**Figure 1:**
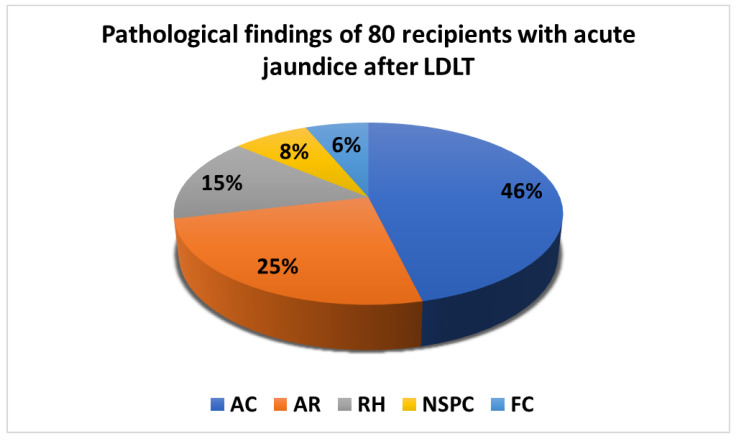
Pathological findings of 80 recipients with acute jaundice after living donor liver transplantation. AC: acute cholangitis; AR: acute cellular rejection; RH: recurrent hepatitis; NSPC: non-specific pathological change; FC: fatty change; LDLT: living donor liver transplantation.

The pathological findings of the 80 recipients undergoing graft biopsy are displayed in Figure 1. The recipients were divided according to the following 5 pathologic findings: acute cholangitis (AC), n = 37, 46%; AR, n = 20, 25%; RH, n = 12, 15%; NSPC, n = 6, 8%; and fatty change (FC), n = 5, 6%. Among the 12 recipients with RH, 4 patients had post-LDLT recurrent hepatitis B, even though their serum HBV DNA was undetectable before and after LDLT, and 8 patients had recurrent hepatitis C concurrently with a high HCV viral load detected in the serum after LDLT. 

Serum liver function test and NLR in peripheral blood samples from the 5 categories are presented in Table 2. No significant difference was observed between the serum biochemistry values and various pathologic results (*p* > 0.05).

### 2.3. Hepatic miRNAs Expression Patterns in Different Graft Pathologies

The expression levels of 4 hepatic miRNAs in different liver graft pathologies are displayed in Table 3. Compared with miRNA-301, miRNA-133a, and miRNA-21, miRNA-122 expression was higher in liver tissue regardless of graft pathological change. Hepatic miRNA-122, miRNA-301, miRNA-133a, and miRNA-21 expression was significantly lower (*p* < 0.05) in AC cases than in RH cases. 

**Figure 2:**
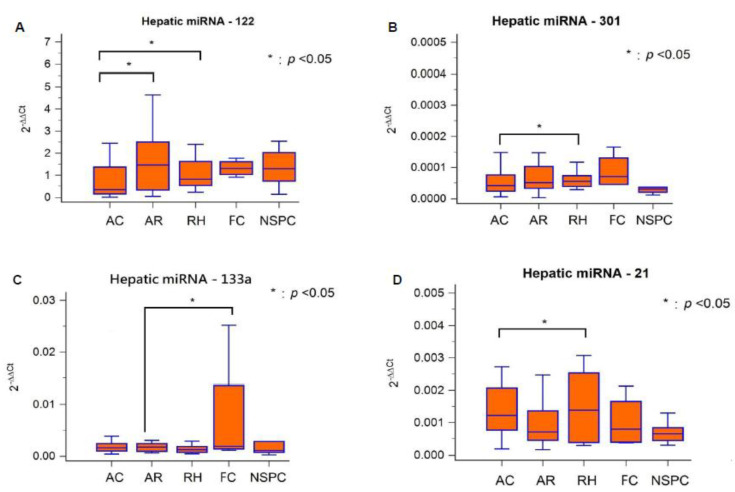
Hepatic micro-RNAs expression level and different liver graft pathologies.

Hepatic miRNA expression levels and different liver graft pathologies in 80 recipients are illustrated separately in Figure 2. Hepatic miRNA-122 expression was significantly high in AR and RH than in AC (*p* < 0.05, Figure 2A); miRNA-301 expression was significantly higher in RH than in AC (*p* < 0.05, Figure 2B); miRNA-133a expression was significantly higher in FC than in AR (*p* < 0.05, Figure 2C); and miRNA-21 expression was significantly higher in RH than in AC (*p* < 0.05, Figure 2D).

To examine the diagnostic performance of the 4 miRNAs as biomarkers for distinguishing different liver graft pathologies, we considered the ROC curves (Supplementary Table S1).

The overall expression patterns of the 4 hepatic miRNAs are displayed as a checklist in Table 4. In AC, miRNA-122, miRNA-301, and miRNA-21 were down-regulated. In AR, miRNA-122 was up-regulated, but miRNA-133a was down-regulated. In RH, miRNA-122, miRNA-301, and miRNA-21 were up-regulated. In FC, only miRNA-133a was up-regulated.

## 3. Discussion

This study evaluated the hepatic miRNA expression patterns, and their potential as biomarkers for distinguishing the etiologies of acute jaundice following LDLT. The main findings of this study are as follows. First, hepatic miRNA-122, miRNA-301, and miRNA-21 expression was significantly lower in AC than in RH. Second, hepatic miRNA-133a expression was higher in FC than in AR. Finally, hepatic miRNA-122 expression was significantly higher in AR than in AC.

The possible reason of the differences and similarities of the upregulation and downregulation of the miRNAs per category might be explained by the organ-specific miRNAs chosen for investigation. In our study, the four miRNAs (miRNA-122, miRNA-301, miRNA-133a and miRNA-21) were all liver-specific miRNAs [14,15,16,17,18]. Both the regulation of miRNAs and pathways (such as TGF-beta signaling pathway and T-Cell, B-Cell, macrophage signaling) involved in liver injury and acute jaundice after living donor liver transplantation were complex [19]; the identification of miRNAs seems to represent only the tip of the iceberg to make definite diagnosis of acute jaundice; however, these liver-specific miRNAs (up-regulation or down-regulation) expression pattern would provide ancillary information other than the histopathology.

In liver transplantation settings, serum biochemistry tests are inadequate for the specific diagnosis of acute jaundice after LDLT, as shown in Table 2. MiRNAs have been reported to be potential biomarkers for liver disease due to their stable nature in samples obtained from tissue or circulating body fluids. Furthermore, miRNAs are tissue-specific and more sensitive to organ damage. In this study, the ROC curve analysis used to evaluate the diagnostic performance of each hepatic miRNA, as a single biomarker for differential diagnosis of liver graft pathologies revealed modest results (Supplementary Table S1). Therefore, combining more than one miRNA expression pattern might provide a more precise prediction for allograft injury, and the present results in checklist form (Table 4) can be employed to help differentiate various pathologic changes. 

In this study, there were 53 (66.3%) recipients who underwent LDLT with primary liver disease of chronic viral hepatitis. Among the 12 recipients with post-LDLT recurrent hepatitis, 4 patients had recurrent hepatitis B, even though their serum HBV DNA was undetectable before and after LDLT; 8 patients had recurrent hepatitis C concurrently with a high HCV viral load detected in the serum after LDLT. According to a previous literature review by Loureiro D et al. [20], cellular miRNAs contribute to HBV and HCV pathogenesis by direct or indirect interactions with viral genome or proteins and molecules critical for the regulation of host or viral genes; the regulation of miRNAs expression upon HBV and HCV infections significantly differs between these viruses. In our study, the result demonstrated that miRNA-122, miRNA-301, and miRNA-21 were remarkably up-regulated in recurrent hepatitis; the discrepancy of miRNA expression levels showed no significance between recurrent hepatitis B and hepatitis C. Further studies are warranted to identify reliable panels of miRNA biomarkers for better differentiation of both.

Currently, histopathologic examination of liver graft biopsy is the gold standard for diagnosing the etiology of acute jaundice, especially for AR or RH; however, liver biopsy is an invasive procedure, and performing repeated or large biopsies for disease monitoring is not feasible. Consequently, information on the alterations and regulatory patterns of specific hepatic miRNAs can be obtained from plasma; the levels of these miRNAs can then be used as optimal diagnostic biomarkers for monitoring the relevant changes before disease progression, thereby facilitating timely treatment [21]. Recently, Thangamani Muthukumar et al. already conduct research using miRNA microarray profiling of RNA from serum matched to liver allograft biopsies from patients with nonimmune, nonviral native liver disease [12]. Their study suggested that quantification of miRNAs in serum using RT-qPCR assays is sufficient for accurate diagnosis of acute rejection or recurrent hepatitis C Virus in human liver allograft recipients; however, different from that study, the innovation of our research is that we focused on hepatic miRNA signatures. As reported in our previous study [13], the miRNA expression level was significantly higher in native liver tissue than in plasma. In this study, we identified miRNAs extracted from native liver tissue, which may represent miRNA expression patterns more accurately than when plasma is used.

Previous studies have confirmed miRNA dysregulation in numerous types of cancer, and miRNA-based therapy for the management of cancers has yielded promising results in the clinical stage [22]. The increasing or decreasing patterns of specific hepatic miRNAs identified in this study may help inform the development of selective miRNA-targeted therapeutics, which could represent the next generation of immunosuppressants for use in liver transplantation [23]. 

Our study has some limitations. First, miRNA expression patterns were not available from healthy recipients to serve as a control group because our liver transplantation program had no protocol for liver graft biopsy. Second, we investigated 4 miRNAs that were hepatocyte-specific and cholangiocyte-specific, but we did not examine immune-cell specific miRNA profiles, which have been reported as tools for detecting allograft rejection [6,21,24,25]. In the future, next-generation sequencing platforms for miRNA sequencing will enable more sensitive detection and exploration of novel miRNAs in clinical samples [7]. Third, the small sample size per category which may not reflect the whole group may be one of the limitations; this would be an important factor why the significant values are minimal (all significance is *p* < 0.05). Fourth, because there was no data available of all miRNAs from serum, it was also one of our limitations in the current study to show the four miRNAs being more accurately and stably expressed in hepatic tissue than circulation. Because there was no data on samples where mixed pathology were found in this study, herein, we were also limited to figure out the reflection of miRNA profiles in mixed pathology. 

In summary, the expression levels of the four intra-hepatic miRNAs are demonstrated in conjunction with liver graft pathology in patients with acute jaundice after LDLT. In acute cholangitis, miRNA-122, miRNA-301, and miRNA-21 were significantly down-regulated; in acute rejection, miRNA-122 was significantly up-regulated and miRNA-133a was significantly down-regulated; in recurrent hepatitis, miRNA-122, miRNA-301, and miRNA-21 were remarkably up-regulated; in fatty change, only miRNA-133a was up-regulated in significance. Consequently, our study revealed that specific intra-graft miRNA expression patterns may serve as ancillary markers for a checklist to help distinguish allograft pathologies in patients with acute jaundice after liver transplantation.

## 4. Methods

### 4.1. Study Population and Design

In this 1-year prospective cohort study, 80 recipients were enrolled in the liver transplantation program at Chang Gung Memorial Hospital. We included only adult patients undergoing liver graft biopsy for the evaluation of post-LDLT acute jaundice (serum total bilirubin > 1.4 mg/dL). The exclusion criteria were as follows: acute issues on chronic liver failure due to sepsis, primary bacterial peritonitis, esophageal variceal bleeding with shock, biliary atresia, pediatric LDLT due to glycogen storage disease, Wilson disease, ABO-incompatible liver transplantation, evidence of hemolytic anemia, and diseased donor liver transplantation.

For patients who were serum positive for HBsAg, pretransplant nucleos(t)ide analogue with entecavir or tenofovir disoproxil fumarate was administered. Hepatitis B immunoglobulin therapy was administered in the anhepatic phase as a single intravenous dose of 10,000 IU; this regimen was continued after LDLT at 2170 IU/day intramuscularly for 7 days, followed by 2170 IU intramuscularly, as needed, to maintain a trough anti-HBs antibody level of >500 IU/l for 3 months, >200 IU/l for 3 to 6 months, and >100 IU/l for >6 months. For patients who were serum positive for HCV RNA, anti-viral therapy was administered using pre-transplant pegylated interferon plus ribavirin or direct-acting antiviral agents.

After LDLT, all recipients received routine serum biochemistry measurements to monitor liver graft function, including on the day of liver graft biopsy. Because protocol liver biopsy after LDLT was not permitted in our liver transplant program, all of the enrolled cases with undetermined etiology of acute jaundice (serum total bilirubin level > 2 mg/dL) after non-invasive image evaluations such as Doppler ultrasound and magnetic resonance cholangiopancreatography were prompted to undergo percutaneous liver graft biopsy. In our study, all of the liver graft biopsy procedures were performed 1–4 weeks after LDLT. Even though both animal (Baker et al., 2015) and human (Tavabie et al., 2021) data demonstrate that miRNA expression is dynamic over time [26,27], the expression level of hepatic micro-RNAs in liver graft would be significantly higher and more accurate than that in serum [13].

All of the 80 recipients undergoing graft biopsy for evaluating the etiology of acute jaundice are divided into 5 groups mainly based on their pathological findings, including acute cholangitis (AC), acute rejection (AR), recurrent hepatitis (RH), non-specific pathological change (NSPC), and fatty change (FC). For histological examination, 5-μm slices of liver samples were stained with hematoxylin and eosin and analyzed by 2 independent liver transplant pathologists. The pathological report was determined according to the diagnostic criteria of histopathology [28]. Acute cholangitis was defined as hepatic parenchyma with cholestasis and neutrophils involving bile duct lumens and epithelium. Acute rejection was defined on the basis of the 1995 Banff classification, and severity grades were characterized according to the rejection activity index. Recurrent hepatitis was mainly HBV or HCV related; the histopathologic hallmark of recurrent hepatitis B was confirmed by the presence of ground-glass hepatocytes that represent HBsAg-containing liver cells; features of recurrent hepatitis C were characterized as lymphoid aggregation in the portal tract and damaged bile duct as well as steatosis [29]. A non-specific pathological change was assumed when no reactive change was observed in the histopathologic examination. Fatty change predominantly diagnosed when macrovesicular steatosis was found in ≥5% hepatocytes [30].

### 4.2. Real-Time Quantitative Reverse Transcription—Polymerase Chain Reaction for Hepatic miRNA Identification in Liver Graft

We evaluated hepatic miRNA levels (miRNA-122, miRNA-301, miRNA-133a and miRNA-21) in liver graft tissues after LDLT. Total RNA was isolated from the liver tissues by using the miRNeasy Mini Kit (Qiagen217004) according to the manufacturer’s protocol. Reverse transcription (RT) was performed with 1 µg RNA using the First-Strand cDNA Synthesis Kit (Promega, Madison, WI, USA) or miScript RT Kit (Qiagen, Hilden, Germany) for the transcription of miRNA according to the manufacturer’s instructions. Using the ABI TaqMan Fast Universal PCR master mix or TaqMan Universal PCR master mix for miRNA (Applied Biosystems, Foster City, CA, USA), we performed RT-PCR on an ABI 7500 Fast Real-Time PCR System with the SDS 1.4 program. The primers and TaqMan MGB probes were obtained from Applied Biosystems, and the final concentrations of primers and probes were 300 and 250 nM, respectively. The cycling profile for each run was 95 °C for 20 s and 40 cycles of 95 °C for 3 s followed by 60 °C for 30 s using the default ramp rate. The results were normalized relative to the levels of glyceraldehyde 3-phosphate dehydrogenase. 

For miRNA profiling, we obtained primers and TaqMan probes for miR-122 (ID: 002245), miR-133a (ID:002246), miR-301 (ID:000528), miR-21 (ID:002438), and U6 small nuclear (snRNA) (ID: 001973) from Applied Biosystems. The cycling profile of each run was 50 °C for 2 min, 95 °C for 10 min, and 40 cycles of 95 °C for 15 s, followed by 60 °C for 1 min using the default ramp rate. The cycle threshold (Ct value), which is inversely correlated with miRNA level, was defined as the number of cycles required for the fluorescent signal to cross the threshold in quantitative PCR. Normalization was performed using U6 snRNA primers. Comparative RT-PCR data, including non-template controls, were obtained in triplicate. The fold increase in cytokine mRNA expression was calculated using the comparative 2^−ΔΔCt^ method, where Ct represents the threshold cycle for each transcript. MicroRNA expression was defined on the basis of Ct. Relative expression levels were calculated as 2^−[(Ct of miR^^-122)^^−(Ct of U6)]^, 2^−[(Ct of miR^^-133a)^^−(Ct of U6)]^, 2^−[(Ct of miR-301)^^−(Ct of U6)]^, and 2^−[(Ct of miR-21)^^−(Ct of U6)]^ after normalization relative to the expression of U6 snRNA [13]. U6 has been shown to be consistently expressed in different tissues and cell types and is widely used for the normalization of cell culture- or tissue-based miRNAs. Differential distribution of U6 expression in human carcinoma tissues demonstrates the requirement for caution in the internal control gene selection for microRNA quantification. Therefore, an accurate determination of miRNA expression levels is fundamental to the elucidation of their biological function. 

### 4.3. Ethical Statement

All procedures involving human participants were performed in accordance with the ethical standards of the institutional committee and with the 1964 Helsinki Declaration and its later amendments or comparable ethical standards. The study protocol was approved and authorized by the Medical Ethics Committee of Chang Gung Memorial Hospital, Kaohsiung (ethical approval number: 201901560B0). We followed the Strengthening the Reporting of Observational Studies in Epidemiology statement guidelines for reporting observational studies. No allograft donor or recipient was from a vulnerable population, and all participants provided written informed consent.

### 4.4. Statistical Analysis

Statistical analyses were performed using SPSS (version 22.0; SPSS Inc., Chicago, IL, USA). Descriptive values are expressed as mean ± standard deviation (SD) and percentages. Categorical variables were compared using the chi-square or Fisher’s exact test, and continuous variables were compared using *Student t*-test. All tests were two-tailed, and a *p* value of <0.05 was considered significant. Receiver operating characteristic (ROC) curves were used to evaluate the relationship between sensitivity and specificity of each hepatic miRNA for differential diagnosis of liver graft pathology.

## Figures and Tables

**Table 1:** Profiles of the 80 recipients with acute jaundice after living donor liver transplantation.

Category	Patients (*n* = 80)
Age, mean ± SD (range) (years)	52.56 ± 13.26 (20–66)
Gender (male/female), n (%)	58 (72.5%)/22 (27.5%)
Primary liver disease, n (%)	
HBV	28 (35%)
HCV	22 (27.5%)
HBV + HCV	3 (3.75%)
Alcohol	14 (17.5%)
Other (Cholangiocarcinoma, Biliary atresia, Primary biliary cirrhosis, Cryptogenic cirrhosis)	13 (16.25%)
Laboratory data	
AFP (ng/dL), mean ± SD	7.29 ± 2.33
Albumin (g/dL)	4.11 ± 0.54
ALT (U/L)	165.19 ± 133.23
AST (U/L)	232.70 ± 194.03
Total bilirubin (mg/dL)	3.25 ± 6.81
Direct bilirubin (mg/dL)	1.91 ± 4.64
ALK-P (U/L)	282.08 ± 335.13
*r*-GT (U/L)	183.17 ± 180.17
WBC (10^3^/μL)	4.48 ± 2.26
Hemoglobin (g/dL)	11.80 ± 3.39
Segment %	62.01 ± 10.87
Lymphocyte %	26.24 ± 10.58
N/L ratio	3.39 ± 2.96

SD: standard deviation; HBV: hepatitis B virus; HCV: hepatitis C virus; AFP: alpha-fetoprotein; ALT: alanine aminotransferase; AST: aspartate transaminase; ALK-P: alkaline phosphatase; γ-GT: γ-glutamyl transferase; N/L ratio: neutrophil/lymphocyte ratio.

**Table 2:** Clinical biochemistry data in different liver graft pathologies after living donor liver transplantation.

Category	AC, n = 37 (46%)	AR, n = 20 (25%)	RH, n = 12 (15%)	FC, n = 5 (6%)	NSPC, n = 6 (8%)	*p* Value
Albumin	4.16 ± 0.53	3.99 ± 0.63	4.03 ± 0.52	4.42 ± 0.45	4.17 ± 0.39	0.140
ALT	152.68 ± 96.75	212.40 ±174.52	152.33 ±187.89	142.00 ± 38.53	130.00 ± 77.21	0.051
AST	217.51 ± 131.87	269.30 ± 183.06	250.50 ± 373.12	167.00 ± 57.29	223.50 ±145.24	0.112
Total Bilirubin	5.71 ± 13.69	5.32 ± 10.40	0.80 ± 0.47	0.90 ± 0.41	0.87 ± 0.37	0.456
Direct Bilirubin	2.20 ± 4.22	10.36 ± 31.55	5.45 ± 17.81	0.24 ± 0.22	0.27 ± 0.12	0.062
ALK-P	375.51 ± 393.50	312.80 ± 339.23	107.50 ± 55.29	99.20 ±44.68	105.00 ± 38.16	0.275
γ-GT	247.81 ± 211.48	165.35 ± 134.16	89.03 ± 104.91	146.00 ±170.66	63.18 ± 53.23	0.060
N/L ratio	3.61 ± 3.76	3.01 ± 2.48	4.46 ± 2.44	1.77 ± 0.77	2.45 ± 0.59	0.227

AC: acute cholangitis; AR: acute cellular rejection; RH: recurrent hepatitis; FC: fatty change; NSPC: non-specific pathological change; N/L ratio: neutrophil/lymphocyte ratio; ALK-P: alkaline phosphatase; γ-GT: γ-glutamyl transferase.

**Table 3:** Comparison of hepatic micro-RNA levels in different liver graft pathologies in 80 recipients who underwent living donor liver transplantation.

Category	miRNA-122	miRNA-301	miRNA-133a	miRNA-21
AC, n = 37 (46%)	1.06 ± 1.39 ^a,b^	0.000068 ± 0.000084 ^c^	0.0086 ± 0.0256	0.00139 ± 0.00101 ^e^
AR, n = 20 (25%)	3.20 ± 7.27 ^a’^	0.000080 ± 0.000079	0.0036 ± 0.0054 ^d^	0.00137 ± 0.00151
RH, n = 12 (15%)	4.05 ± 10.37 ^b’^	0.000189 ± 0.000430 ^c’^	0.0159 ± 0.0465	0.00319 ± 0.00608 ^e’^
FC, n = 5 (6%)	1.09 ± 0.60	0.000071 ± 0.000061	0.0402 ± 0.0738 ^d’^	0.00087 ± 0.00079
NSPC, n = 6 (8%)	1.34 ± 0.78	0.000050 ± 0.000061	0.0039 ± 0.0067	0.00070 ± 0.00035

AC: acute cholangitis; AR: acute rejection; RH: recurrent hepatitis; NSPC: non-specific pathological change; FC: fatty change. Two-tailed Student’s *t*-test was conducted for comparison of micro-RNAs levels; a *p-*value of < 0.05 means statistically significant. ^a:a’^, *p* < 0.05; ^b:b’^, *p* < 0.05; ^c:c’^, *p* = 0.05; ^d:d’^, *p* < 0.05; ^e:e’^, *p* < 0.05. As labeled in Table 3, “^a^” and “^b^” were miRNA-122 expression level in AC; “^a’^” was miRNA-122 expression level in AR; “^b’^” was miRNA-122 expression level in RH; “^c^” was miRNA-301 expression level in AC; “^c’^” was miRNA-301 expression level in RH; “^d^” was miRNA-133a expression level in AR; “^d’^” was miRNA-133a expression level in FC; “^e^” was miRNA-21 expression level in AC; “^e’^” was miRNA-21 expression level in RH.

**Table 4:** Liver graft pathologies and hepatic micro-RNAs expression patterns in acute jaundice after living donor liver transplantation.

Category	miRNA-122	miRNA-301	miRNA-133a	miRNA-21
Acute cholangitis (AC)	▽	▽		▽
Acute rejection (AR)	▲		▽	
Recurrent hepatitis (RH)	▲	▲		▲
Fatty change (FC)			▲	

▲: up-regulation; ▽: down-regulation.

## Data Availability

Because of the participant consent obtained as part of the recruitment process, it is not possible to make these data publicly available. The data resented in this study are available on request from the corresponding author.

## Supplementary Materials

The following are available online at https://www.mdpi.com/article/10.3390/biology11081228/s1, Table S1: Receiver operating characteristic (ROC) curves for evaluation of each 4 miRNA hepatic miRNAs (miRNA-122, miRNA-301, miRNA-133a and miRNA-21) as biomarkers of different liver graft pathology.
