# Supplementary material for: Liver Graft MicroRNAs Expression in Different Etiology of Acute Jaundice after Living Donor Liver Transplantation
Source: Biology (Basel). 2022 Aug 17;11(8):1228. doi: 10.3390/biology11081228 (PMC9404977; doi:10.3390/biology11081228)
Supplement: Supplementary file 1 [file biology-11-01228-s001.zip › biology-1872911-supplementary.pdf]

Supplement Table S1. Receiver operating characteristic (ROC) curves for evaluation of each 4 miRNA hepatic miRNAs (miRNA-122, miRNA-301, miRNA-133a and miRNA-21) as biomarkers of different liver graft pathology.

miRNA -122

<table>
<tr><td>AC</td><td>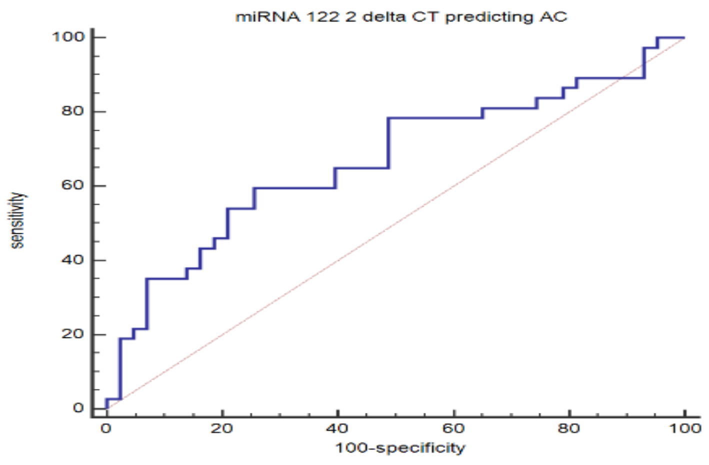
</td><td>cutoff value: <= 0.5948
AUC = 0.668
P = 0.008*
sensitivity = 59.46
specificity = 74.42
95% CI = 0.553-0.769
Youden J = 0.3388</td></tr>
<tr><td>AR</td><td>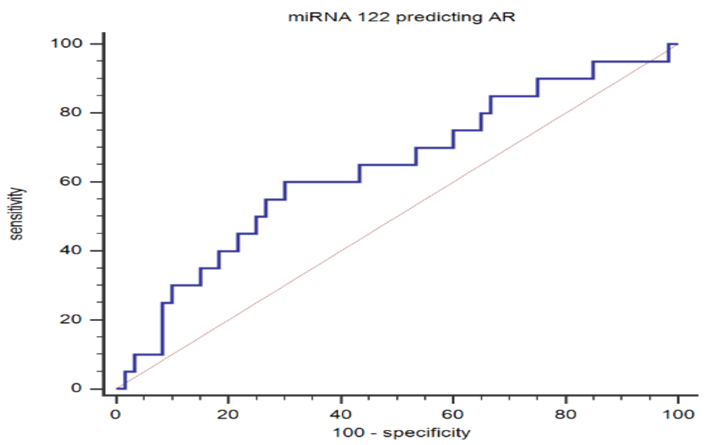
</td><td>cutoff value: > 1.4337
AUC = 0.638
P = 0.0652
sensitivity = 60.0
specificity = 70.0
95% CI = 0.523 - 0.743
Youden J = 0.3000</td></tr>
<tr><td>RH</td><td>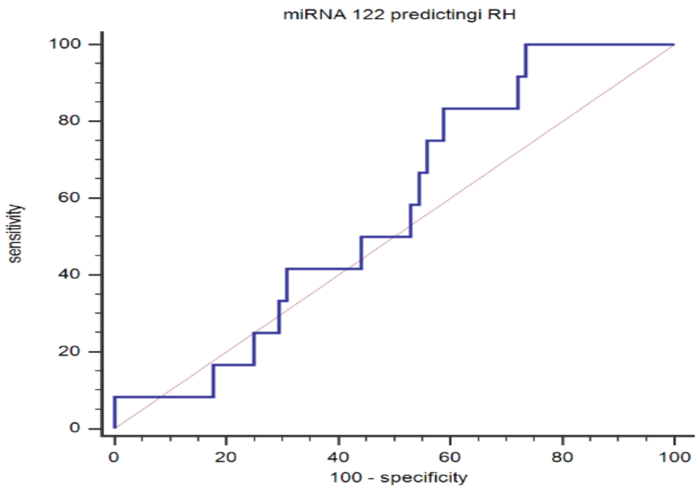
</td><td>cutoff value: > 0.2117
AUC = 0.571
P = 0.3614
sensitivity = 100.0
specificity = 26.47
95% CI = 0.455 - 0.681
Youden J = 0.2647</td></tr>
</table>

**FC**	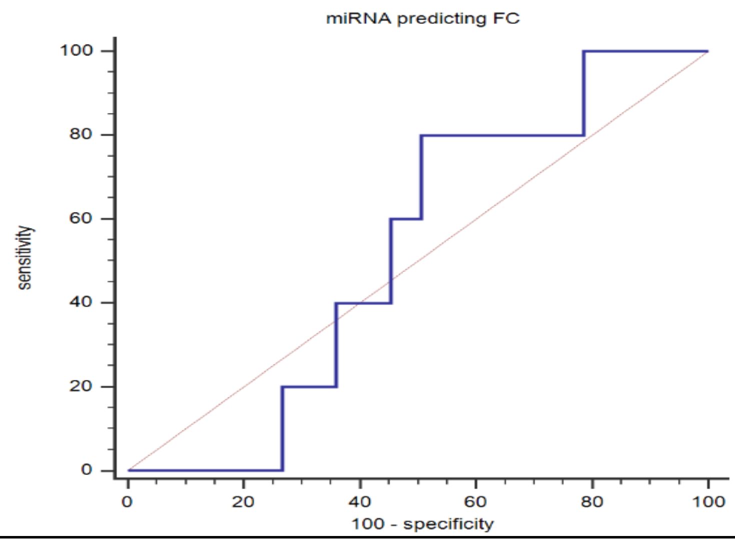	cutoff value: > 0.8249<br>AUC = 0.525<br>P = 0.7985<br>sensitivity = 80.00<br>specificity = 49.33<br>95% CI = 0.411 - 0.638<br>Youden J = 0.2933
**NSPC**	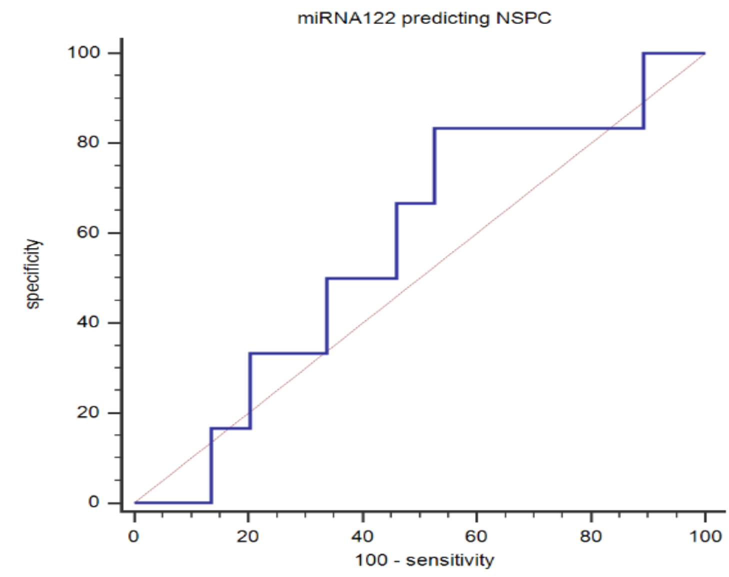	cutoff value: > 0.6268<br>AUC = 0.574<br>P = 0.5276<br>sensitivity = 82.33<br>specificity = 47.30<br>95% CI = 0.459 - 0.684<br>Youden J = 0.3063

miRNA -301

<table>
<tr>
<td>AC</td>
<td>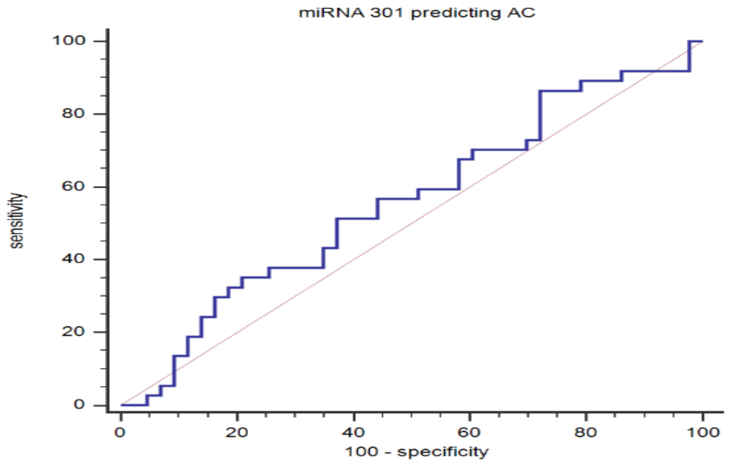
</td>
<td>cutoff value: <=0.00008
AUC = 0.559
P = 0.3689
sensitivity = 86.49
specificity = 27.91
95% CI = 0.443 - 0.670
Youden J = 0.1439</td>
</tr>
<tr>
<td>AR</td>
<td>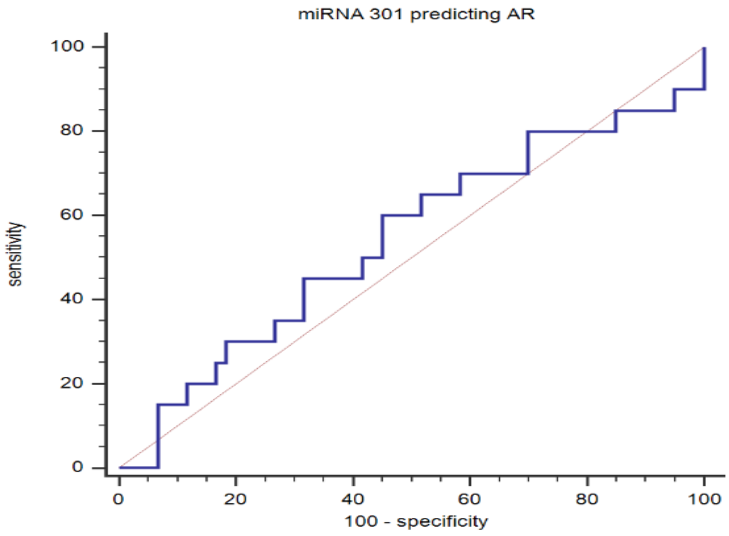
</td>
<td>cutoff value: >0.00005
AUC = 0.541
P = 0.6072
sensitivity = 60.00
specificity = 55.00
95% CI = 0.426 - 0.653
Youden J = 0.1500</td>
</tr>
<tr>
<td>RH</td>
<td>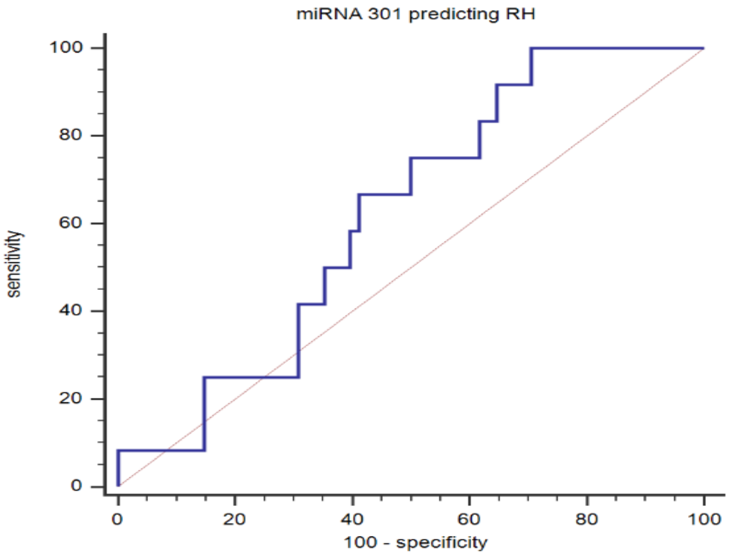
</td>
<td>cutoff value: >0.00003
AUC = 0.621
P = 0.1066
sensitivity = 100.00
specificity = 29.41
95% CI = 0.506 - 0.727
Youden J = 0.2941</td>
</tr>
</table>

**FC**	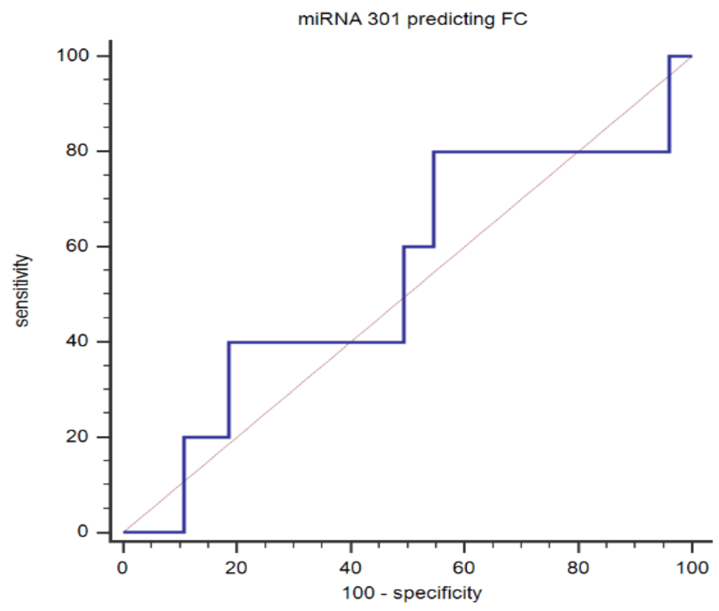	cutoff value: > 0.00004<br>AUC = 0.541<br>P = 0.7897<br>sensitivity = 80.00<br>specificity = 45.33<br>95% CI = 0.426 - 0.653<br>Youden J = 0.2533
**NSPC**	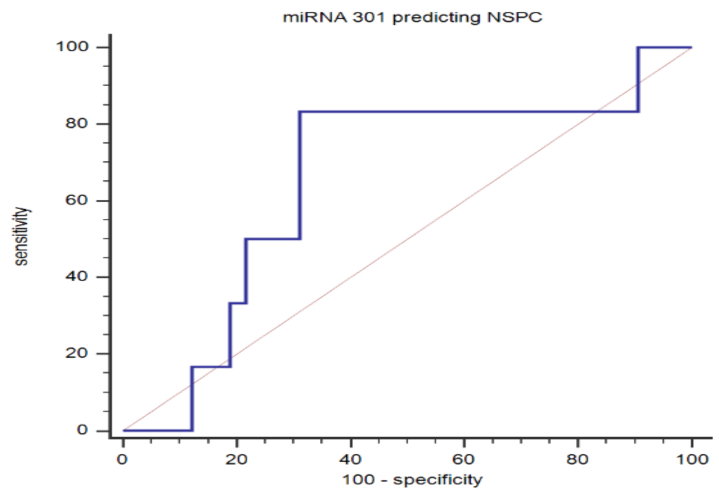	cutoff value: <=0.00004<br>AUC = 0.658<br>P = 0.1978<br>sensitivity = 83.33<br>specificity = 68.92<br>95% CI = 0.543 - 0.760<br>Youden J = 0.5225

miRNA -133a

<table>
<tr><td>AC</td><td>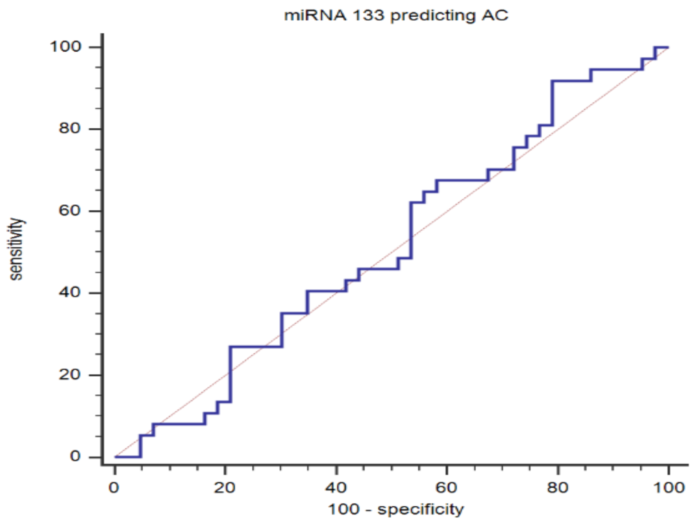
</td><td>cutoff value: >0.0007
AUC = 0.515
P = 0.8140
sensitivity = 91.89
specificity = 20.93
95% CI = 0.401 - 0.629
Youden J = 0.1282</td></tr>
<tr><td>AR</td><td>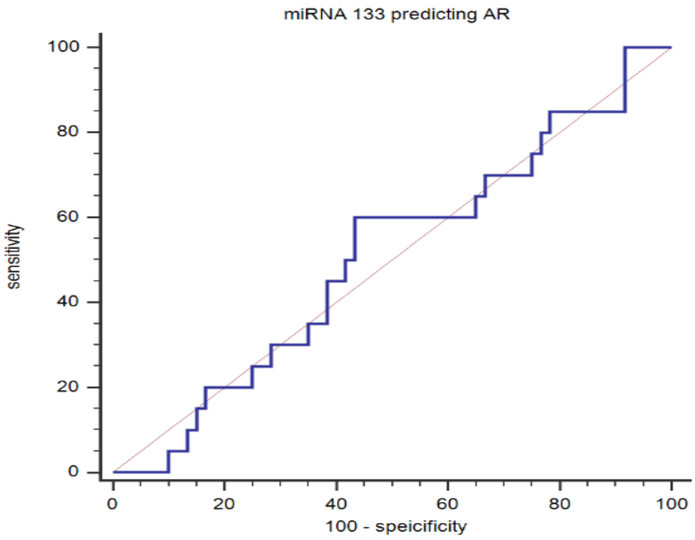
</td><td>cutoff value: >0.0017
AUC = 0.508
P = 0.9196
sensitivity = 60.00
specificity = 56.67
95% CI = 0.393 - 0.621
Youden J = 0.1667</td></tr>
<tr><td>RH</td><td>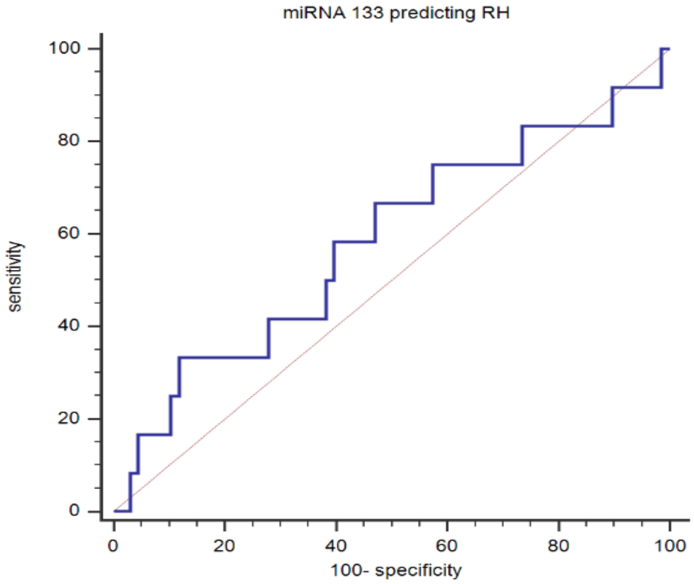
</td><td>cutoff value: <=0.0007
AUC = 0.582
P = 0.4099
sensitivity = 33.33
specificity = 88.24*
95% CI = 0.466 - 0.691
Youden J = 0.2157</td></tr>
</table>

**FC**	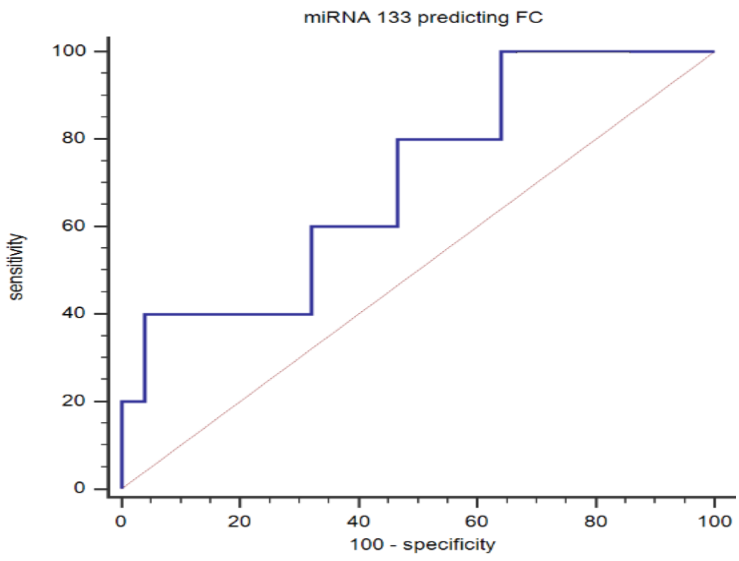	cutoff value: >0.00011<br>AUC = 0.707<br>P = 0.1024<br>sensitivity = 100.0<br>specificity = 36.0<br>95% CI = 0.594 - 0.803<br>Youden J = 0.3600
**NSPC**	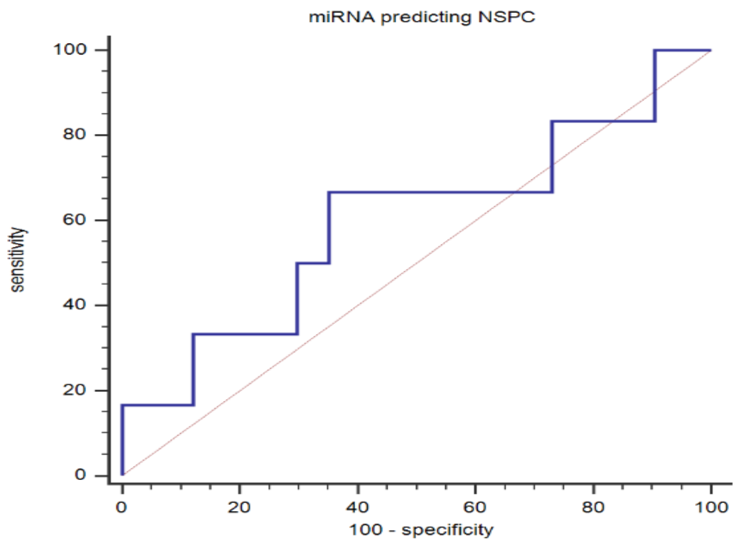	cutoff value: <=0.0012<br>AUC = 0.599<br>P = 0.4975<br>sensitivity = 66.67<br>specificity = 64.86<br>95% CI = 0.483 - 0.707<br>Youden J = 0.3153

miRNA -21

**AC**	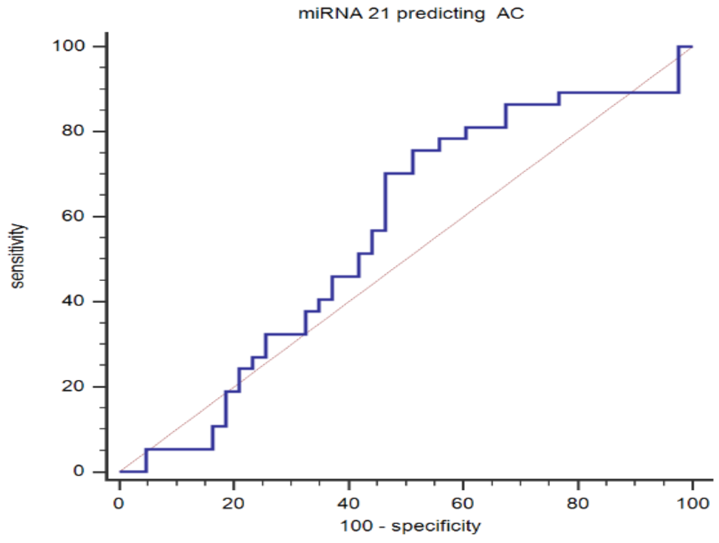	cutoff value: > 1.4337<br>AUC = 0.638<br>P = 0.0652<br>sensitivity = 60.0<br>specificity = 70.0<br>95% CI = 0.523 - 0.743<br>Youden J = 0.3000
**AR**	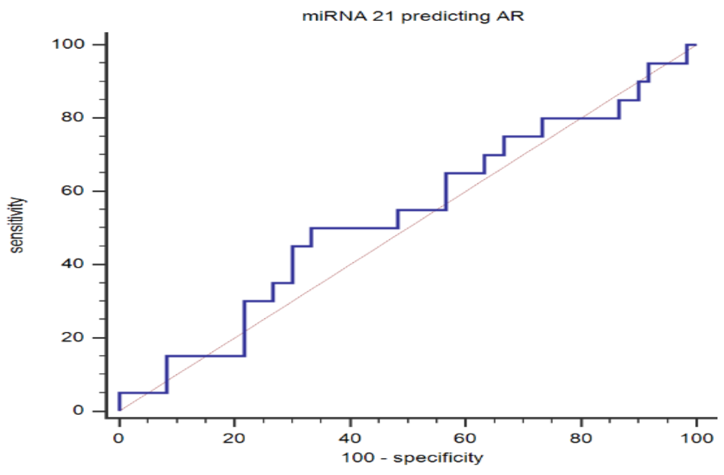	cutoff value: >0.0007<br>AUC = 0.533<br>P = 0.0777<br>sensitivity = 50.0<br>specificity = 66.67<br>95% CI = 0.418 - 0.646<br>Youden J = 0.1667
**RH**	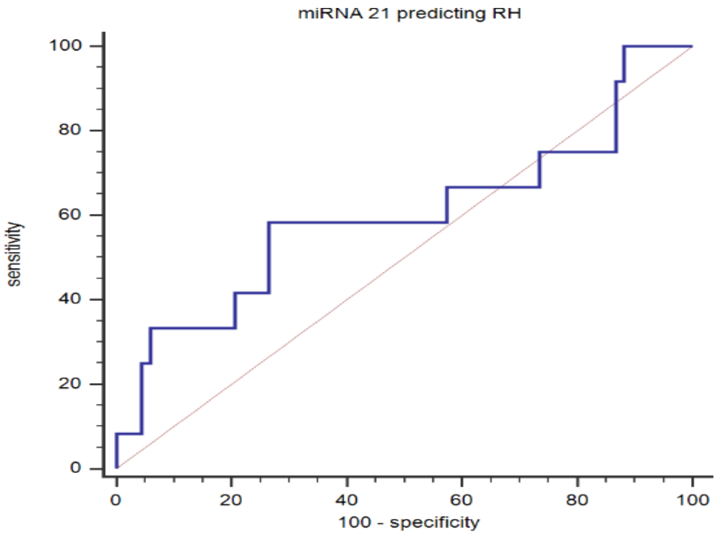	cutoff value: >0.0014<br>AUC = 0.599<br>P = 0.3542<br>sensitivity = 58.33<br>specificity = 73.53<br>95% CI = 0.484 - 0.707<br>Youden J = 0.3186

**FC**	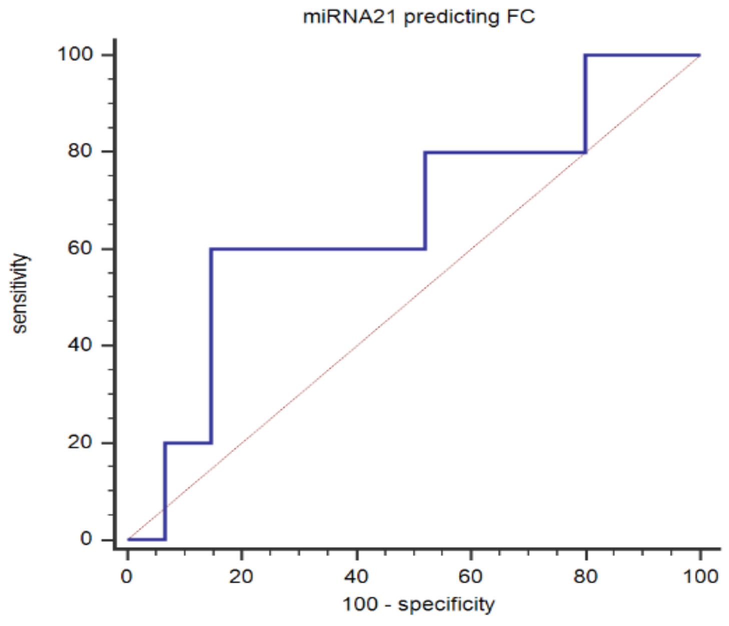	cutoff value: <=0.000425745<br>AUC = 0.664<br>P = 0.2543<br>sensitivity = 60.00<br>specificity = 85.33<br>95% CI = 0.550 - 0.766<br>Youden J = 0.4533
**NSPC**	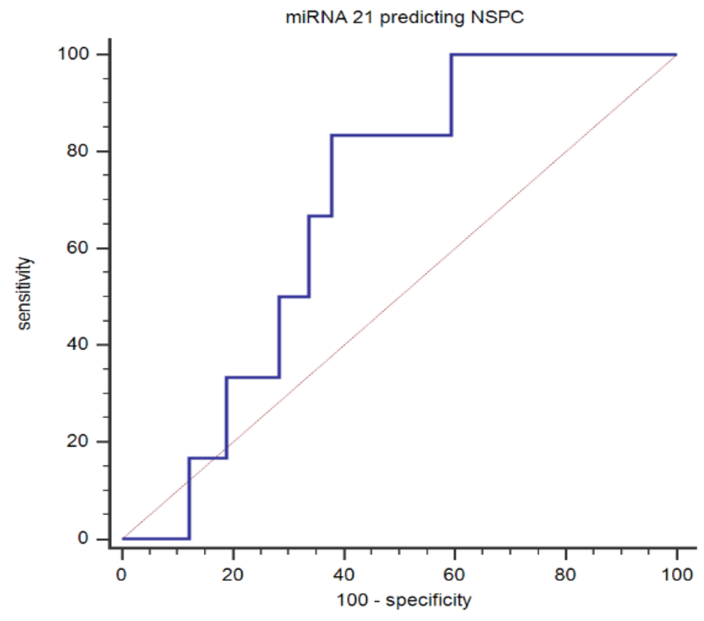	cutoff value: <=0.0008<br>AUC = 0.682<br>P = 0.0225*<br>sensitivity = 83.33<br>specificity = 62.16<br>95% CI = 0.569 -0.782<br>Youden J = 0.4550
